# Does Resveratrol Play a Role in Decreasing the Inflammation Associated with Contrast Induced Nephropathy in Rat Model?

**DOI:** 10.3390/jcm8020147

**Published:** 2019-01-27

**Authors:** Yi-Hsin Chen, Yun-Ching Fu, Ming-Ju Wu

**Affiliations:** 1 Institute of Clinical Medicine, National Yang-Ming University, Taipei 112, Taiwan; nephp06@gmail.com (Y.-H.C.); yunchingfu@gmail.com (Y.-C.F.); 2 Department of Nephrology, Taichung Tzu Chi Hospital, Buddhist Tzu Chi Medical Foundation, Taichung 427, Taiwan; 3 School of Medicine, Tzu Chi University, Hualien 907, Taiwan; 4 School of Medicine, College of Medicine, China Medical University, Taichung 404, Taiwan; 5 Section of Pediatric Cardiology, Department of Pediatrics, Taichung Veterans General Hospital, Taichung 407, Taiwan; 6 Division of Nephrology, Department of Internal Medicine, Taichung Veterans General Hospital, Taichung 407, Taiwan; 7 School of Medicine, Chung Shan Medical University, Taichung 402, Taiwan

**Keywords:** contrast induced nephropathy, resveratrol, inflammasome

## Abstract

Contrast is widely used in invasive image examinations such as computed tomography (CT) and angiography; however, the risk of contrast-induced nephropathy (CIN) is high. The aim of this study was to investigate the protective effect of resveratrol in a rat model of CIN. Sprague-Dawley rats were divided into four groups: the control group (0.9% saline infusion only); resveratrol group (RSV, resveratrol, 30 mg/kg); contrast media group (CIN); and resveratrol + contrast media group (RCIN, resveratrol 30 mg/kg 60 min before CIN). CIN was induced via an intravenous injection of a single dose of indomethacin (10 mg/kg), one dose of N-nitro-L-arginine methyl ester (10 mg/kg), and a single dose of contrast medium iopromide (2 g/kg). Blood urea nitrogen, creatinine, and neutrophil gelatinase-associated lipocalin (NGAL) were higher in the CIN group compared to the other groups. Histopathological tubule injury scores were also higher in the CIN group compared to the other groups (*p* < 0.01). NLPR3 in kidney tissue were higher in the CIN group compared to the other groups; however, these results were improved by resveratrol in the RCIN group compared with the CIN group. The expressions of IL-1β and the percentage of apoptotic cells were higher in the CIN group than in the control and RSV groups, but they were lower in the RCIN group than in the CIN group. The expression of cleaved caspase-3 was higher in the CIN group than in the control and RSV groups, but lower in the RCIN group than in the CIN group. Resveratrol treatment attenuated both injury processes and apoptosis and inhibited the inflammasome pathway in this rat CIN model.

## 1. Introduction

Contrast-induced nephropathy (CIN) is an important cause of acute kidney injury [1], and it has been significantly associated with comorbidity and mortality in hospitalized patients with acute kidney injury after contrast examinations or coronary angiography [2]. Fluid resuscitation has been reported to play a role in renal protection; however, it cannot prevent acute kidney injury completely, especially in patients with chronic kidney disease [3]. 

The mechanism of CIN has yet to be fully elucidated [4]. Renal hypoxia and the subsequent generation of reactive oxygen species, vasoconstriction, and inflammation have all been reported to play a central role in the pathogenesis of CIN [5]. Previous studies have also indicated that inflammatory cells and cytokines are involved in the mechanisms of CIN [6,7]. Interactions between leukocytes and endothelium can result in the activation of inflammatory responses and further decrease the microcirculatory perfusion of the outer medulla [8,9]. NLPR3 (Pyrin Domain Containing 3) inflammasome is involved in tissue injury under infectious and non-infectious conditions, and it has attracted increasing interest. Fang at el. reported that caspase-1, IL-1β, and IL-18 were significantly up-regulated in patients with proteinuria [10], and Zhuang et al. showed that inhibiting NLRP3 via siRNA remarkably attenuated albumin-induced cell apoptosis and phenotypic changes under both in vitro and in vivo conditions [11]. Shen et al. demonstrated the role of NLRP3 inflammasome in mediating CIN through modulating the apoptotic pathway [12].

Resveratrol (3,5,4′-trihydroxystilbene) is a natural polyphenol found in grapes and red wine, and it has been reported to possess anti-inflammatory effects in previous investigations [13]. Resveratrol can activate the mammalian sirtuin Sirt1 [14]. Mammalian Sirt1 deacetylates a host of target proteins that are important for apoptosis, the cell cycle, circadian rhythms, mitochondrial function, and metabolism [15,16,17,18,19]. Administration of resveratrol can exert a cytoprotective effect and prevent kidney disease, cardiovascular disease, and cancer through Sirt1 [17,20,21]. The severity of diabetic nephropathy can be attenuated by resveratrol through anti-oxidative and anti-inflammatory effects [22]. Iopromide, a low osmolality, non-ionic, radiocontrast agent, can induce renal tubular cell apoptosis and nephropathy [23,24]. Previous animal studies have reported that resveratrol can ameliorate chronic kidney disease and acute kidney injury [13], and it was also shown to decrease the inflammatory cells in the kidney, such as polymorphonuclear granulocytes, in acute kidney injury models in previous work [25]. 

The aim of this study was to investigate the role of resveratrol in mediating anti-inflammatory responses and its potential therapeutic role in CIN.

## 2. Materials and Methods

### 2.1. Animals

The male Sprague-Dawley (SD) rats used in this study weighed 250–300 g (2 months old) and were purchased from the National Laboratory Animals Center (Taipei, Taiwan). The rats were raised in standard cages with strictly controlled temperature (21–25 °C), humidity (30–50%), and automatic lighting control (12 h light/12 h dark). We used metabolic cages to monitor the fluid and food intake in 24 h until rats were sacrificed. We performed experimental procedures complying with animal care guidelines, and the Animal Care and Research Committee of Taichung Veterans General Hospital approved this protocol (permit number: La-101982).

### 2.2. Experimental Design

All experiments were performed in accordance with the Institutional Animal Care and Use Committee. The animals (*n* = 32) were randomly divided into four groups of eight rats each. The rats in the control group received only saline injections into the femoral vein, while those in the resveratrol (RSV) group received an injection of resveratrol (30 mg/kg; Sigma-Aldrich) into the femoral vein. The dosage of resveratrol was according to previous studies [26,27]. The rats in the CIN group underwent experimental induction of CIN via the intravenous administration of indomethacin (10 mg/kg), N-nitro-L-arginine methyl ester (10 mg/kg, after 15 min), and iopromide (2 g/kg; Bayer HealthCare Pharmaceuticals, Berlin, Germany), as previously described [28,29,30,31,32,33]. The vasodilatory effects of prostaglandins may have countered the vasoconstriction caused by contrast media. Pretreatment with indomethacin was necessary to induce contrast induced renal injury in the rat model [34]. The rats in the resveratrol plus contrast media (RCIN) group received resveratrol (30 mg/kg) via the femoral vein 60 min before induction of CIN during the experiment. All rats were allowed to recover in metabolic cages for 24 h and were then sacrificed by rapid decapitation. Blood samples were obtained from the abdominal aorta. Serum was separated and aliquots were stored at −80 °C until analysis. 

### 2.3. Renal Function and Cytokine Analysis

For evaluating renal function, serum urea, and creatinine were measured using ELISA kits (Sigma, Saint Louis, MO, USA) using the manufacturer’s protocols. Frozen kidney tissues were homogenized in lysis buffer (150 mM NaCl, 15 mM Tris, 1 mM MgCl_2_ pH 7.4, 1 mM CaCl_2_, 1% Triton) with a 1% protease inhibitor cocktail (P8340, Sigma, Saint Louis, USA). The procedures were executed according to the manufacturer’s instructions.

### 2.4. Histopathology and Immunochemistry

Renal tissues were put in 10% buffered formalin overnight and subsequently embedded in paraffin. Renal sections of 4-μm thickness were stained with hematoxylin and eosin. Vacuolar degeneration of kidney tubular cells was counted and scored as follows: less than 5% = 0, 5–20% = 1+, 20–50% = 2+, and more than 50% = 3+. The scoring method was executed according to previous studies [35,36]. The sections were evaluated under a light microscope by a technical assistant (J.Q.H.) in a masked manner.

Renal cell apoptosis was evaluated on 4-μm renal sections using a Terminal deoxynucleotidyl transferase (TdT) deoxyuridine triphosphate nick end labeling (TUNEL) assay by DeadEnd Colorimetric TUNEL System (Promega, Madison, USA) according to the manufacturer’s instructions. TUNEL-positive stained renal cells were counted in 10 fields selected randomly in each slide, and the data were presented as the percentage of apoptotic cells per field.

### 2.5. Western Blot

The expressions of NLR family pyrin domain containing 3 (NLRP3), IL-1β and cleaved caspase-3 were analyzed using Western blotting. Proteins were extracted from kidney tissues with radioimmunoprecipitation assay (RIPA) buffer, which contained protease inhibitors (PMSF) and sodium orthovanadate. Protein (50 μg) was isolated by SDS-PAGE and transferred onto a PVDF membrane using a wet transfer apparatus. The blots were blocked with 5% nonfat dry milk in TBS. Later, they were incubated with primary antibodies at 4 °C overnight. The samples were washed and then treated with horseradish peroxidase (HRP) labeled secondary antibodies at 4 °C. After 2 h, the protein was detected using Pierce ECL Western Blotting Substrate (Pierce, Rockford, IL, USA). Primary antibodies against NLRP3 (R&D Systems, Abingdon, UK), cleaved caspase-3 (Cell Signaling Technologies, Denver, MA, USA), IL-1β (Abcam, Cambridge, UK), and β-actin (Sigma, Saint Louis, USA) were used. The secondary antibody was HRP-conjugated anti-IgG (Jackson ImmunoResearch Laboratories, Inc., West Grove, PA, USA). The immunoreactive bands were presented using a Western BLoT HRP Chemiluminescent Substrate system. We use β-actin as an internal control.

### 2.6. Enzyme-Linked Immunosorbent Assay for Plasma Neutrophil Gelatinase-Associated Lipocalin

Serum neutrophil gelatinase-associated lipocalin (NGAL) can be used as a good predictive biomarker in acute kidney injury [37]. Microtiter plates pre-coated with a goat polyclonal antibody along with rat NGAL were treated in blocking buffer containing 1% BSA, and then coated with 100-μL samples of plasma (ranging from 1–200 ng/mL). The plates were then treated with a horseradish peroxidase conjugated, affinity purified rabbit anti-goat IgG antibody, and a TMB substrate was used for color development. The data was acquired after the plates were put within 30 min (at 450 nm) under a Benchmark Plus microplate reader (Bio-Tek Instruments Inc., Winooski, VT, USA).

### 2.7. Data Analysis

Data were presented as mean ± standard error. Group comparisons were evaluated by ANOVA followed by Tukey’s test. A *p* value < 0.05 was considered to be significant. All analyses were performed using Graphpad Prism 6.0 software (Graphpad Software, Inc., La Jolla, CA, USA).

## 3. Results

### 3.1. Resveratrol Attenuated Renal Injury and Protected Renal Function after the Induction of Contrast Nephropathy

**Figure 1:**
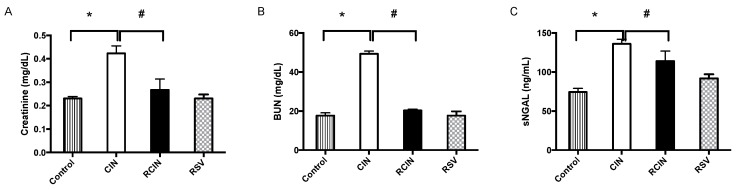
Resveratrol attenuated renal injury after exposure to contrast medium. Blood of the control, contrast-induced nephropathy (CIN), resveratrol + contrast media (RCIN), and resveratrol (RSV) groups of rats were collected 24 h after exposure to contrast medium. Serum (**A**) creatinine, (**B**) BUN, (**C**) Serum neutrophil gelatinase-associated lipocalin (NGAL). Data are presented as mean ± standard error (*n* = 8 per group). *: *p* < 0.05 versus control; #: *p* < 0.05 versus RCIN. RSV = resveratrol; CIN = contrast-induced nephropathy; RCIN = resveratrol before contrast-induced nephropathy. Group comparisons were evaluated by ANOVA followed by Tukey’s test.

Serum creatinine and blood urea nitrogen (BUN) were used as markers of renal dysfunction after CIN had been induced. The markers were significantly higher in the CIN group compared with the control group (Figure 1A,B), indicating markedly impaired kidney function after the induction of CIN in our model. The RCIN group had a 37.1% recovery in the serum level of creatinine (Figure 1A) and a 58.8% recovery in the serum level of BUN (Figure 1B) compared with the CIN group. Resveratrol did not affect the serum NGAL levels in the RSV group compared to the control group (Figure 1C). However, exposure to contrast induced the upregulation of serum NGAL levels by 83.1% in the CIN group, and this effect was attenuated by 16.4% by resveratrol in the RCIN group (Figure 1C).

### 3.2. Resveratrol Preserved the Renal Architecture after the Induction of Contrast Nephropathy

**Figure 2:**
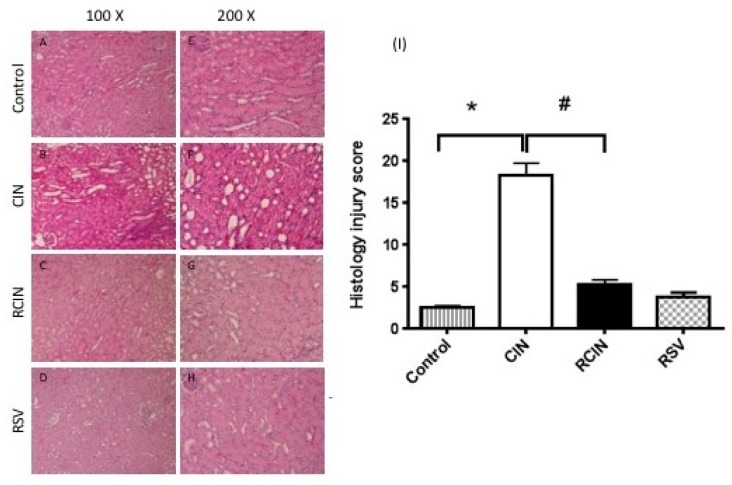
Histopathological findings of rat kidney after CIN had been induced. Kidney tissues of the CIN and RCIN rats were collected 24 h after contrast nephropathy had been induced. Representative photomicrographs of histologic staining with hematoxylin and eosin in the corticomedullary junction at ×100 (left, **A–D**) and ×200 magnification (right, **E–H**). (**A** and **E**) Control rats. (**B** and **F**) Rats treated with contrast. (**C** and **G**) Rats treated with contrast after resveratrol treatment. (**D** and **H**) Control rats pretreated with resveratrol. RSV = resveratrol; CIN = contrast-induced nephropathy; RCIN = resveratrol before contrast-induced nephropathy. (**I**) Histologic scores of contrast nephropathy. Histopathology scoring was assessed in a blinded fashion. The scoring system reflecting the grading of tubular necrosis, loss of brush border, cast formation, and tubular dilatation in 10 randomly chosen, non-overlapping fields (200×) was as follows: 1, <10%; 2+, 10–25%; 3+, 26–75%; and 4+, >75%. The score in the CIN group was 18.3 ± 3.4, compared with 2.5 ± 0.5 in the control group. Treatment with resveratrol (RCIN group) before contrast significantly reduced the histologic injury score to 5.2 ± 1.5 versus RCIN. All statistical analyses were performed by Kruskal-Wallis test, followed by Dunn’s multiple comparison post hoc test (*n* = 8 for each group). *: *p* < 0.05 versus control; #: *p* < 0.05 versus RCIN.

The CIN group presented marked differences in renal histology when compared with the control group. The most severe lesions were observed in the renal tubules. The lesions included severe vacuolar changes in the cytoplasm, tubular cast formation, and congestion of tubular lumen (Figure 2B,F). However, no obvious glomerular change was seen in the CIN group. Kidney sections from the RCIN group demonstrated markedly improved histological features of each of these above renal tubular insults (Figure 2C,G).

### 3.3. Resveratrol Attenuated Renal Injury Scores after Contrast Nephropathy Had Been Induced

The extent of kidney injury was further graded in the four groups (Figure 2I). The differences in injury were semiquantitatively represented. The scores ranged from 0–20, with a higher number representing a more severe injury. The scores were 18.3 ± 3.4 in the CIN group, 2.5 ± 0.5 in the control group, and 5.2 ± 1.5 in the RCIN group (Figure 2I).

### 3.4. Effects of Resveratrol on Renal Tubular Apoptosis after Contrast Nephropathy Had Been Induced

**Figure 3:**
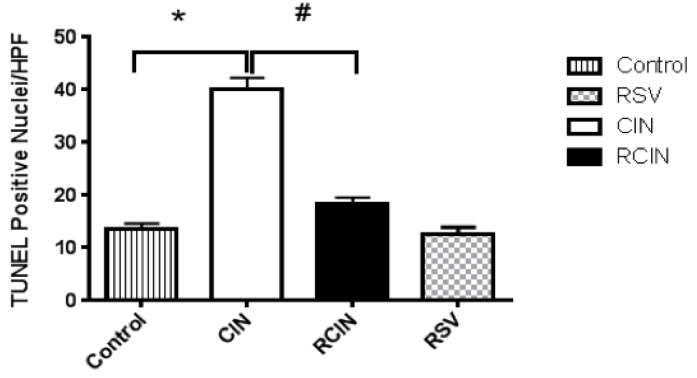
Numeric data of apoptotic renal nuclei are presented. Quantitative analyses of TUNEL-positive cells revealed that pretreatment with resveratrol significantly decreased the number of apoptotic cells by 54.3% in the RCIN group. RSV = resveratrol; CIN = contrast-induced nephropathy; RCIN = resveratrol before contrast-induced nephropathy. All statistical analyses were performed by Kruskal-Wallis test, followed by Dunn’s multiple comparison post hoc test (*n* = 8 for each group). *: *p* < 0.05 versus control; #: *p* < 0.05 versus RCIN.

Evaluation of apoptotic renal tubular cells using terminal deoxynucleotidyl transferase–mediated deoxyuridine triphosphate nick-end labeling (TUNEL) immunostaining to localize DNA fragmentation in kidney tissues showed that the kidneys from the CIN group had a marked increase in the number of TUNEL-positive tubular cells, but the count was markedly less in the RCIN group. Measurement of TUNEL-positive cells also disclosed that pretreatment with resveratrol significantly decreased the number of apoptotic cells by 54.3% in the RCIN group (Figure 3).

### 3.5. Resveratrol Pretreatment Ameliorated Kidney Inflammation by Blocking NLRP3 Inflammasome Activation after Contrast Nephropathy Had Been Induced

**Figure 4:**
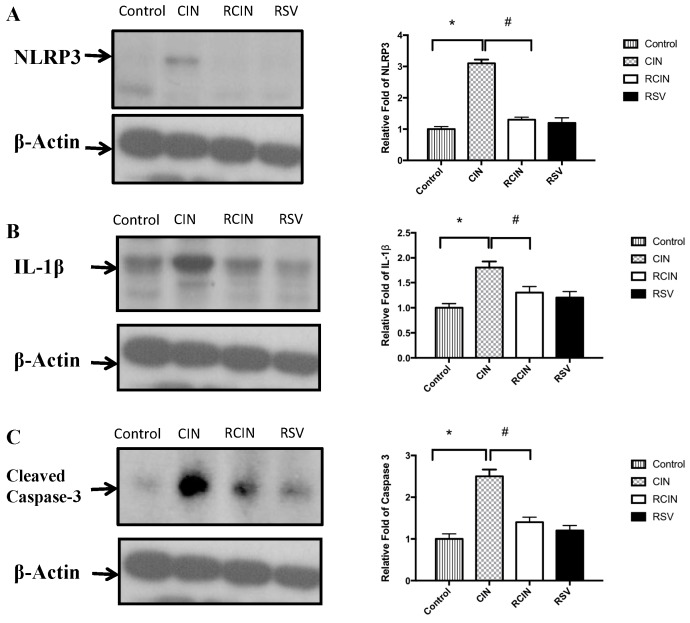
Western blot analysis of renal NLRP3, IL-1β, and cleaved caspase-3. The expression of renal (**A**) NLRP3, (**B**) IL-1β, and (**C**) cleaved caspase-3 increased after the injection of contrast medium in the CIN group, as assessed by Western blot analysis. Resveratrol significantly suppressed the expressions of cytokines in the RCIN group. *, #: *p* < 0.05 comparing the two groups. NLRP3 = NLR Family Pyrin Domain Containing 3, CIN: contrast-induced nephropathy, RCIN: pretreatment with resveratrol before CIN, RSV: resveratrol 30 mg/kg iv only.

Western blot analyses demonstrated that the expression levels of renal NLRP3 were increased in the CIN group compared with the control group (Figure 4A). The overexpression of renal NLRP3 in the CIN group was dramatically decreased by pretreatment with resveratrol in the RCIN group. 

### 3.6. Resveratrol Pretreatment Attenuated the Expression of Proinflammatory Cytokines in the Kidney after Contrast Nephropathy Had Been Induced

As shown in Figure 4B,C, the expressions of kidney IL-1β and cleaved caspase-3 increased after exposure to contrast media in the CIN group, as assessed by Western blot analysis. Resveratrol significantly suppressed cytokine expression in the RCIN group. 

## 4. Discussion

In this study, we found that resveratrol attenuated acute kidney injury in a rat model of CIN, and also suppressed renal tubular injuries. Injection with contrast agent iopromide and pretreatment with N-nitro-L-arginine methyl ester and indomethacin produced renal dysfunction, which is consistent with previous CIN studies in rodent models [38,39]. 

The direct toxic effect of contrast medium on renal tubular cells has been reported to play a key role in developing acute kidney injury [40]. In the current study, we found pathological changes in renal tubular cells including cytoplasmic vacuolar changes, tubular necrosis, and features of contrast medium-induced kidney injury. We also found that resveratrol protected against these cytotoxic effects, as evidenced by a significant decrease of 54.3% in the number of apoptotic cells in the RCIN group when comparing to CIN group (Figure 3).

Recent studies have proposed that resveratrol is also an anti-inflammatory and anti-aging agent [41,42,43], exerting neuroprotective roles through autophagy in cellular model [19]. In Sener’s study, resveratrol provided anti-fibrotic effect in the rat model of bleomycin-induced pulmonary fibrosis [44]. Further studies related to resveratrol involving autophagy and fibrosis in CIN model could be explored in the future.

NGAL has been reported to be a potentially useful marker of sustained renal injury after acute kidney injury [45]. Therefore, we used serum levels of NGAL as a biomarker of renal injury in our CIN model. Serum NGAL concentrations have been reported to be powerful independent predictors of CIN [46,47]. In addition, NGAL was also reported to be a good marker of acute kidney injury due to hemorrhagic shock in a previous animal models [48]. Elevations in NGAL levels precede changes in serum creatinine and can be used to diagnose acute kidney injury up to 48 h prior to a clinical change in creatinine or urine output. In our model, serum levels of NGAL in CIN group were increased by 83.1% compared to the control group, and suppressed by 16.4% when RSV was given before injections of the contrast medium. 

NLRP3 inflammasome has also been shown to be a mediator of ischemic acute kidney injury, as evidenced in a model of NLRP3 knockout mice which were protected from acute kidney injury [49]. NLRP3 activation induces three types of caspase-1-mediated responses: secretion of IL-1β, secretion of IL-18, and a programmed form of cell death, referred to as pyroptosis [50]. Several studies have demonstrated that NLRP3-related danger signaling triggers renal inflammation including post-ischemic or oxalate crystal-induced and in immune complex glomerulonephritis [51,52]. Sirt1 exerts anti-inflammatory effects by regulating the expression of NLRP3 [53]. As resveratrol is a Sirt1 activator, it suppresses NLRP3 inflammasome activation by preserving mitochondrial integrity and augmenting autophagy [54]. Our data also provide evidence that resveratrol can attenuate NLRP3 inflammasome in a CIN model, as the level of renal NLRP3 in our CIN group was dramatically reduced by pretreatment with resveratrol in the RCIN group. Previous studies had indicated that NLRP3 may provide a potential therapeutic target for the treatment of contrast media induced acute kidney injury [5,12]. Hong et al. demonstrated the protective effect of resveratrol in mice model for CIN via SIRT1-PGC-1α-Foxo1 pathway [55]. Their result revealed recovery of Sirt1 and PGC-1α and improvement of renal apoptosis after resveratrol administration in CIN model. Our study may complement their study from point view of NLRP3 inflammasome.

Resveratrol pretreatment has been reported to attenuate caspase-3 and -8 activation and prolong cell survival after UVB irradiation [56]. IL-1β is one of the main proinflammatory cytokines that regulates a broad range of immune responses and also participates in several physiological processes [57]. Many chronic inflammatory diseases are related to the overproduction of IL-1β, and IL-1β is mediated by the NLRP3 inflammasome [58]. Ionizing radiation-induced IL-1β secretion by human mesenchymal stem cells has also been reported, and that this was effectively abrogated by pretreatment with resveratrol [53]. In other words, radiation-induced increases in IL-1β occur via activation of NLRP3 inflammasome, and resveratrol treatment attenuates the expression of NLRP3. Resveratrol treatment has also been shown to upregulate the expression of Sirt1, and IL-1β has been shown to be a proximal mediator of the inflammatory events associated with infection, sepsis, and ischemia [59]. Renal levels of IL-1β, IL-18, and IL-6 and neutrophils have been reported to be higher in cisplatin-induced acute kidney injury [59]. Expression of cleaved caspase-3 is a marker of cellular apoptosis. A previous study by He et al. showed a decrease of cleaved caspase-3 fragments when Sirt1 is activated, protecting from oxidative injury in the mouse renal medulla [16]. In our study, resveratrol decreased the expression of NLRP3 in the RCIN group and hence attenuated the expressions of IL-1β and caspase-3. 

Our study only used eight rats in each group with a total of thirty-two. This is a limitation of our study. Future studies may expand the number of rats.

## 5. Conclusions

Our results demonstrate that resveratrol can inhibit apoptosis and inflammasome expression induced in a CIN model. Previous studies have shown that resveratrol can suppress NLRP3 and subsequent IL-1β in sepsis models [60]. Hence, the regulation of NLRP3 inflammasome in our acute kidney injury model conferred protection from subsequent kidney injury. These findings may be helpful when developing renoprotective drugs before the use of contrast medium.
